# Effect of Different Running Exercise Modalities on Post-Exercise Oxidative Stress Markers in Trained Athletes

**DOI:** 10.3390/ijerph17103729

**Published:** 2020-05-25

**Authors:** Wajdi Souissi, Mohamed Amine Bouzid, Mohamed Amine Farjallah, Lobna Ben Mahmoud, Mariem Boudaya, Florian A. Engel, Zouheir Sahnoun

**Affiliations:** 1 Research Laboratory: Education, Motricité, Sport et Santé, EM2S, LR19JS01, High Institute of Sport and Physical Education, University of Sfax, Sfax 3000, Tunisia; souissi.wajdi@gmail.com (W.S.); bouzid.mohamed-amine@hotmail.fr (M.A.B.); aminefarjallah@hotmail.fr (M.A.F.); 2 Research Center on Sport and Movement (Centre de Recherchessur le Sport et le Mouvement, CeRSM), UPL, Université Paris Nanterre, UFR STAPS, F-92000 Nanterre, France; 3 Pharmacology Department, Faculty of Medicine, University of Sfax, Sfax 3000, Tunisia; benmahmoud_lobna@medecinesfax.org (L.B.M.); zouheir.sahnoun.fms@gmail.com (Z.S.); 4 Biochemistry Laboratory, CHU HediChaker, University of Sfax, Sfax 3000, Tunisia; Mariem.boudaya@yahoo.fr; 5 Institute of Sport and Sport Science, Heidelberg University, 69120 Heidelberg, Germany; 6 Department of Sport Science, Bundeswehr University Munich, 85577 Neubiberg, Germany

**Keywords:** oxidative stress, exercise, athletes, free radical damages, antioxidant defenses

## Abstract

The aim of this study was to examine the effect of running exercise modality on oxidative stress. Thirteen endurance athletes (age: 21.46 ± 0.66 years) performed three different running exercise modalities (Continuous running exercise (CR): continuous running exercise at 75% of VO_2max_ for 25 min; intermittent running exercise #1 (15/15): intermittent running protocol, 15 s running at 75% of VO_2max_, 15 s passive recovery, performed for 50 min; intermittent running exercise #2 (30/30): intermittent running protocol, 30 s running at 75% of VO_2max_, 30 s passive recovery, performed for 50 min) in a randomized order. Blood samples were drawn at rest and immediately after each running exercise and assessed for malondialdehyde (MDA), advanced oxidation protein products (AOPP), superoxide dismutase(SOD), and glutathione peroxidase (GPX) activities. MDA increased by 55% following 30/30 exercise (*p* < 0.01), while it remained unchanged with CR and15/15 exercise. SOD increased after CR (+13.9%, *p* < 0.05), and also remained unchanged after 15/15 (*p* > 0.05) and decreased after 30/30 (−19.7% *p* < 0.05). GPX and AOPP did not change after exercise in all experimental sessions (*p* > 0.05). In conclusion, 30/30 intermittent running induced higher lipid damages than the 15/15 and CR exercise. 15/15 intermittent exercise promoted a better balance between free radicals production and antioxidant defense compared to continuous exercise and intermittent 30/30 exercise.

## 1. Introduction

The practice of regular physical activity [1] and running [2] are recognized as essential factors for maintaining good health (e.g., fighting against cardiovascular diseases, osteoarthritis, diabetes, and osteoporosis) [1]. However, the practice of long and/or intense physical exercise can expose athletes to muscle injuries and chronic fatigue, which can be directly linked to the toxic effects of free radicals (FR) [3].

Oxidative stress is an imbalance between the biochemical processes of FR production and antioxidant defenses [4]. Classically, elevated levels of oxidative damage markers, likemalondialdehyde (MDA) and advanced oxidation levels of the protein products (AOPP) are associated with increased oxidative stress, while elevated levels of antioxidants, such as superoxide dismutase (SOD) and glutathione peroxidase (GXP) are associated with decreased oxidative stress. When the redox equilibrium or homeostasis is disrupted, cells become vulnerable to free radical attack, resulting in oxidative damage to cellular components [5]. FR are widely recognized for their dual roles as being both deleterious and beneficial, since they can be either harmful or beneficial to living systems, particularly by playing a physiological role in intracellular signaling and regulation as secondary messengers of the expression of antioxidant enzymes, such as SOD, and improves oxidation resistance [6]. However, under certain conditions, FR produced during exercise may exceed the body’s antioxidant capacity and contribute to muscle fatigue [7], inflammation, and tissue damage caused by the oxidation of macromolecules [8].

Physical exercise affects FR production and the antioxidant capacity that could contribute to a disruption of the balance between these two entities. Many studies focused on sports, involving aerobic metabolism, like running or swimming [9,10,11], showed an increase in FR production of, as well as an increase in the activity of antioxidant enzymes, such as SOD, GPX, and catalase (CAT). In several studies, changes in oxidant/antioxidant balance could be explained by an increase in oxygen consumption during exercise [12,13,14]. Indeed, the increase in oxygen consumption during exercise promotes a large leakage of FR in the mitochondria, and then results in an antioxidant reaction.

In endurance training sessions, coaches and athletes adopt two types of exercise: either continuous exercise or intermittent exercise. Although these two types of exercise do promote the development of the aerobic capacity, their solicitation of energy metabolism is not the same. Indeed, Combes et al. [15] reported higher oxygen consumption during continuous exercise compared to intermittent exercise, consisting of 30 s of running, interspersed with 30 s passive recovery. The same authors in another study compared different types of intermittent exercise (i.e., 30/30 s, 60/60 s, and 120/120 s) and showed that oxygen consumption increases proportionally with the duration of intermittent exercise [16]. As a result, this variation in oxygen consumption could potentially have different effects on the production of FR and FR damage in athletes. 

The impact of the exercise modality (continuous vs. intermittent) on oxidative stress is an ongoing debate. Numerous studies compared the exercise modalities with the same energy expenditure and different exercise durations, and reported that both modality and duration of the exercise could impact the oxygen consumptiondifferently [17,18,19]. As a result, the comparison of variable exercise load (intermittent) vs. constant exercise load (continuous) with the same overall energy expenditure and the same exercise duration would clearly identify the effect of the exercise modality on oxidative stress.

Therefore, the present study aims to determine the type of exercise (continuous vs. intermittent) allowing a minimal level of oxidative stress. The results will allow a better understanding of oxidative stress responses to different modalities of aerobic running exercise. It would improve the prescription of endurance running training by targeting training modalities favoring less radical damage, in order to preserve the athlete’s health and to optimize the process of recovery after training.

Therefore, the aim of this study wasto investigate the effects of different running exercise modalities (continuous, intermittent 30/30 s, and intermittent 15/15 s) on antioxidant defenses and markers of radical damage in male athletes. 

We hypothesized that continuous exercise could lead to moreradical damage compared to intermittent exercise, and that intermittent exercise 15/15 could lead to higher antioxidant defenses.

## 2. Materials and Methods 

### 2.1. Participants

Thirteen male athletes (Mean ± standard deviation: age: 21.46 ± 0.66 years, weight: 76.62 ± 7.53 kg, height: 1.79 ± 0.08 m) voluntarily participated in this study. The inclusion criteria were: (i) training a minimum of 8 h per week; (ii) healthy; (iii) does not consume alcohol and caffeine. After receiving a full description of the study protocol and the possible risks and benefits associated with the study, each participant signed a written informed consent form prior to participation. The study was conducted in accordance with the Declaration of Helsinki [20]. Additionally, the protocol was fully approved by the Research Ethics Committee of the High Institute of Sport and Physical Education of Sfax, University of Sfax, Tunisia before the commencement of the assessments.

### 2.2. Experimental Protocol

All participants completed an initial session to ensure familiarization with all measures and procedures. Athletes performed a time-to-exhaustion test (VAMEVAL) 72 h following the familiarization session in order to determine their maximal aerobic running speed (MAS). VAMEVAL is a triangular test in which participants exercise to exhaustion. The test starts at 8.5 km·h^−1^, and the increment of speed was 0.5 km·h^−1^ every 60 s. Thereafter, and in counterbalanced design, participants performed the three different running exercise modalities in three different experimental sessions, separated by three days of recovery for all participants. Onesessionconsists of a continuous running exercise (CR) at 75% of MAS for 25 min. In another session, athletes performed an intermittent running exercise #1 (15/15) consisting of 15 s of running at 75% of MAS, interspersed by 15 s of passive recovery, performed for 50 min. The third session consisted of an intermittent running exercise #2 (30/30) in which participants performed an intermittent running protocol, consisting of 30 s of running at 75% of MAS interspersed by 30 s of passive recovery, performed for 50 min.

**Figure 1:**
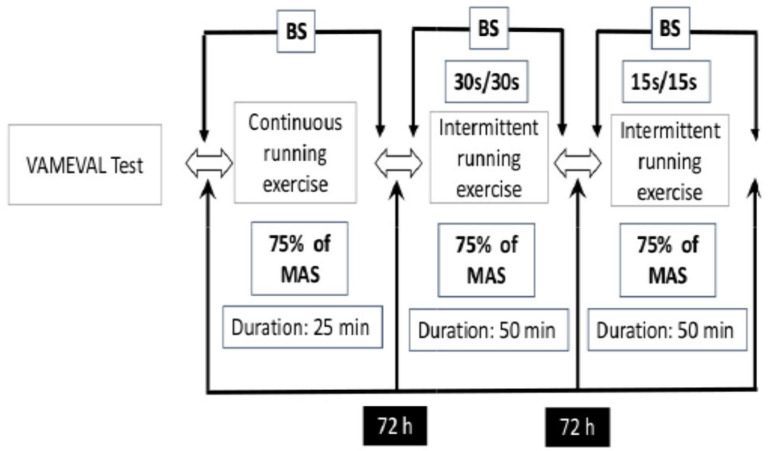
Protocol design. BS: blood sample; MAS: maximal aerobic speed.

The three running protocols were performed outdoors on a PVC running surface. Pacing was controlled using time emitted for each 50 m running distance. In order to limit the influence of exogenous factors on oxidative stress parameters and on running performance, written indications were given to each participant before the commencement of the experiment—three days prior to each experimental session, no training sessions were allowed and participants were requested to refrain from any recovery treatments (e.g., massage, compression garments, cold water immersion). The participants were asked to refrain from consuming any alcoholic or caffeinated beverages 24 h prior to each experimental session and to maintain their normal dietary habits for the duration of the study. Finally, heart rate (HR) was recorded for each experimental session (Polar Team2 Pro, Polar, Finland). The experimental protocol is represented in Figure 1. Each testing session was performed at the same time of day to minimize the diurnal variation effect on performance (between 4:30 p.m. and 6:30 p.m.).

### 2.3. VAMEVAL Test

For assessment of maximum aerobic speed (MAS), participants performed the VAMEVAL field test [21]. It is an incremental running test performed on a 400 m outdoor running track. Participants were required to run between markers set 20 m apart at varying speeds dictated by an audio signal. The test started with 8.5 km/h^−1^, and the running speed increased by 0.5 km/h^−1^ every minute until voluntary exhaustion of the participant. The test ended when participants were not able to maintain the required running speed dictated by the audio signal at two consecutive occasions. MAS was considered as the highest running velocity maintained throughout a complete stage during the VAMEVAL test.

### 2.4. Dietary Records

During the period, the three different running exercise modalities were performed, and participants recorded food intake. At the first visit tothe laboratory, a standardized individual information session was performed to instruct subjects to record their daily food intake. Food quantities were estimated, specifying the number of units and a code corresponding to the size of the portion, using a reference portion guideline book. Data collected from each participant was analyzed using the Bilnut 4 software package (SCDA Nutrisoft, Cerelles, France) and the food composition tables published by the Tunisian National Institute of Statistics in 1978 [22].

### 2.5. Blood Sampling and Analysis

Blood samples (5 mL of blood) were taken from an antecubital vein at rest and immediately after each running exercise. After the blood samples were drawn, samples were immediately centrifuged at 3000 rpm at a temperature of 4 °C for 10 min. Then, the plasma obtained was divided into 8 tubes and frozen at −80 °C. Subsequently to defreezing, the blood samples were analyzed for MDA, AOPP, SOD, and GPX. Blood was collected in EDTA, cut off from oxygen and light. Hematocrit and hemoglobin were measured as part of a complete blood count using an automated cell counter (Coulter LH 750; Beckman Coulter, Brea, CA, USA). Finally, plasma volume was then corrected using the guidelines provided by Dill and Costill [23].

### 2.6. Protein Rate Determination

Total protein concentration was determined by the Bradford method [24], calibrated with bovine serum albumin.

### 2.7. Antioxidants Measurement (SOD)

According to Beauchamp and Fridovich [25], SOD activity was evaluated for its ability to inhibit photochemical reduction of nitrobluetetrazolium (NBT). The reaction mixture contained 0.1 M potassium phosphate buffer (pH 7.4), 0.26 mM riboflavin, 2.69 mM methionine, and 2.64 mM NBT, with a plasma suitably diluted in a total volume of 1.5 mL. The assay mixture was illuminated for 20 min with a 20 W fluorescent lamp in an aluminum lined container. Reduction of NBT by the blue-colored formazan superoxide radicals was monitored at 580 nm. SOD activity was expressed in U/mg protein.

### 2.8. GPX

GPX activity was quantified by the procedure of Flohé and Günzler [26]. The plasma was added to a reaction mixture containing 0.1 M potassium phosphate buffer (pH 7.4) and 4 mM GSH. H_2_O_2_ (5 mM) was added, then mixed after 10 min of incubation at 37 °C. TCA (5%) stopped the reaction. After centrifugation at 3000 rpm for 10 min at 4 °C, the supernatant was combined with phosphate buffer and 10 mM DTNB and the absorbance read at 412 nm. GPx activity was expressed in nmol GSH consumed/min/mg protein.

### 2.9. Markers of Radical Damages (MDA)

MDA is the principal and most studied product of polyunsaturated fatty acid peroxidation. It is also one of the most popular markers used to evaluate oxidative stress damage on lipids in the literature in view of the facility and accessibility of its detection in comparison with other markers of lipid peroxidation, like F2-isoprostanes and lipid hydroperoxyde. MDA was assessed by thiobarbituric acid (TBA) reactive substances by measuring plasma levels of MDA, using the method described by Buege and Aust [27]. Samples were mixed with TBA solution (15% trichloroacetic acid [TCA], 0.8% TBA, 0.25 N HCl), then incubated at 95 °C for 15 min. The mixture was then centrifuged at 3000 rpm for 10 min and cooled in ice for 5 min. Absorption of supernatants was read at a wavelength of 532 nm. Concentrations were reported as nmol MDA/mg protein.

### 2.10. AOPP

The method of Kayali et al. [28] was used to determine the advanced oxidation levels of the protein products (AOPP). Plasma was treated with phosphate buffer (0.1 M, pH 7.4). After 2 min of incubation, 1.16 Mpotassiumiodide and 10% of TCA were added to the mixture. The AOPP concentration for each sample was calculated based on an extinction coefficient of 261 cm-1mM-1 at 340 nm and expressed in nmol/mg protein.

### 2.11. Statistical Analysis

All data are presented as means ± standard deviation (SD), and were analyzed using STATISTICA for Windows software (version 6.0, StatSoft, Inc, Tulsa, OK, USA). The normality of every dependent variable and homogeneity of the variances of the distributions (equal variance) were confirmed using the Shapiro–Wilk test and the Levene test, respectively. For all physical and biochemical data, a two-way ANOVA with repeated measures [exercise (continuous, 15/15, 30/30) × time (pre-exercise vs. post-exercise)] was used. When appropriate, post hoc comparisons were made with the Bonferroni test. Statistical significance was accepted at *p* < 0.05.

## 3. Results

### 3.1. Physiological Parameters

Heart rate values were significantly higher in 15/15 compared to CR (168 ± 11 bpm and 146 ± 12 bpm respectively) (*p* < 0.05). However, no significant differences were revealed between heart rate values during 30/30 (155 ± 8 bpm) and the two other running exercise protocols (*p* > 0.05).

### 3.2. Dietary Intake

No significant differences in dietary intake were reported between the three experimental sessions (Table 1).

### 3.3. MDA Level

Statistical analysis revealed a significant interaction modality × time (F = 9.69; *p* < 0.01; ɳp^2^ = 0.51). The post hoc test showed a significant increase of MDA concentration immediately after 30/30 intermittent running, compared to the resting values (+55.05 % ± 8.5) (*p* < 0.01).

**Figure 2:**
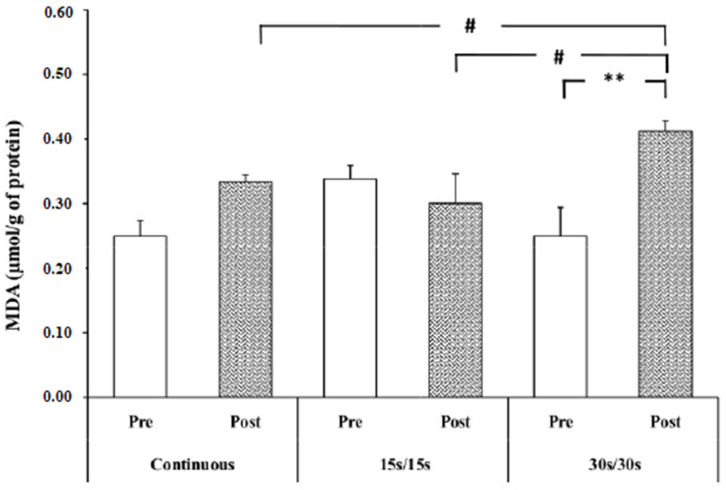
MDA level at rest and immediately after exercise. **: significantly different from resting values (*p* < 0.01); #: significantly different from 30/30 intermittent exercise (*p* < 0.05).

In addition, post-exercise MDA levels in 30/30 intermittent running were significantly higher compared to CR and 15/15 intermittent running (*p* < 0.05) (Figure 2).

### 3.4. AOPP Level

Statistical analysis revealed only an effect of exercise modality (F = 5.90; *p* < 0.01, ɳp^2^ = 0.37) on AOPP level. Following the exercise, AOPP did not change in CR (+10.3 ± 3.2), 15/15 (+6.7 ± 2.5) and in the 30/30 intermittent running exercise (+11.6 ± 4.5) (*p* > 0.05). In addition, statistical analysis showed that exercise-induced variation was similar between the three experimental trials (*p* = 0.73).

**Figure 3:**
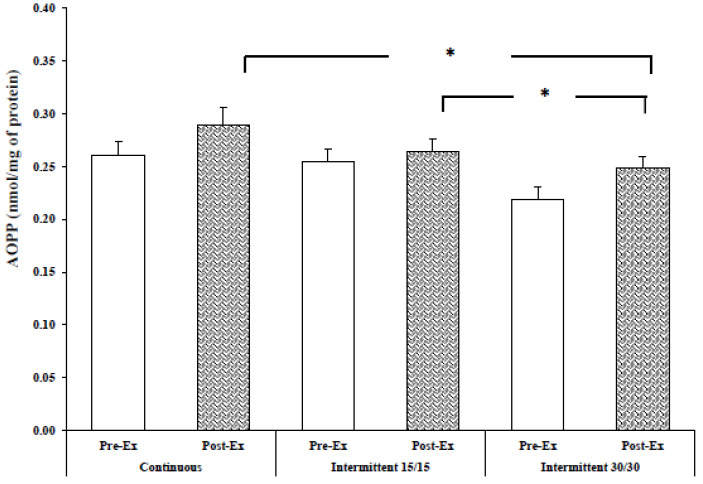
AOPP level at rest and immediately after exercise. #: significantly different from 30/30 intermittent exercise (*p* < 0.05).

Nevertheless, we noted that post-exercise AOPP concentration was significantly higher in CR compared to 30/30 intermittent running (*p* < 0.05). Likewise, AOPP levels following the 15/15 intermittent running exercise was significantly higher compared to 30/30 intermittent running (Figure 3).

### 3.5. SOD Activity

Statistical analysis showed a significant modality × time interaction (F= 7.01; *p* < 0.01, ɳp^2^ = 0.41) for SOD values. The post hoc test showed a significant increase of SOD concentration after CR compared to the resting value (+13.9 %) (*p* < 0.05).

**Figure 4:**
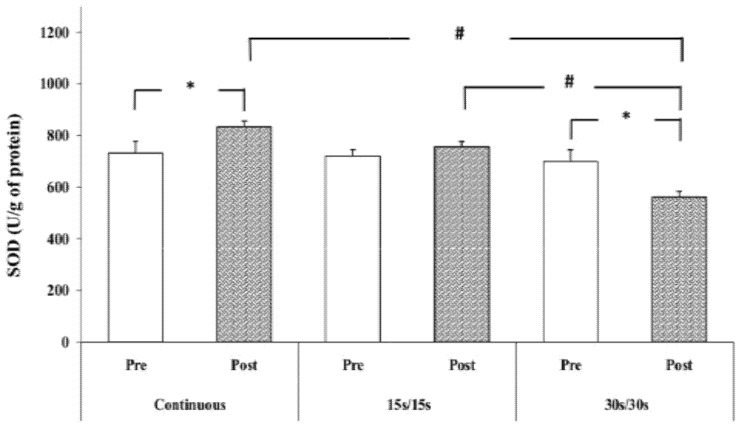
SOD activity at rest and immediately after exercise. *: significantly different from resting values (*p* < 0.05); #: significantly different from 30/30 intermittent exercise (*p* < 0.05).

However, following the 30/30 running protocol, a significant decrease (*p* < 0.05) of SOD concentration was observed compared to the resting value (−19.77 %). Following the 15/15 running protocol, the SOD concentration remained unaltered. In addition, the post hoc test revealed that post-exercise SOD concentration was significantly lower in 30/30 compared to CR and 15/15 intermittent running protocols (560.9 ± 83.03 U/g protein; 833.63 ± 80.42 U/g protein and 756.44 ± 75.55 U/g protein, respectively) (*p* < 0.01) (Figure 4).

### 3.6. GPX Activity

**Figure 5:**
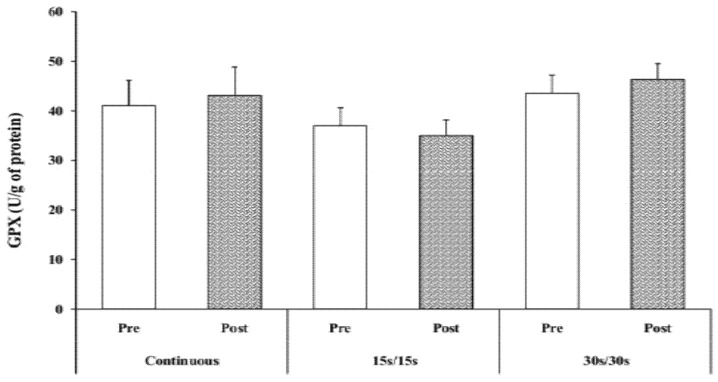
GPX activity at rest and immediately after exercise.

Statistical analysis did not reveal any significant effects of time or exercise modality on GPX activity (*p* > 0.05). Additionally, no significant interactions (modality × time) were observed (Figure 5).

## 4. Discussion

The aim of the present study was to analyze the effects of different running exercise modalities on antioxidant and free radical damages in male athletes. The main finding of the present study wasthat following the running exercise, oxidative stress parameters (MDA, AOPP, SOD) respondeddifferently depending on the running exercise modality. In fact, MDA level and AOPP level following CR exercise and 15/15 intermittent running exercise were higher compared to 30/30 intermittent running exercise. SOD increased after CR exercise, decreased following 30/30 intermittent running exercise, and remained unchanged after 15/15 intermittent running exercise. However, GPX did not change after exercise in all experimental sessions.

These findings might have implications for understanding the oxidative stress responses to different running exercise modalities, which may improve the prescription of running training by targeting training modalities leading to minimal free radical damage, in order to preserve the health of athletes by optimizing the process of recovery following running training.

In the present study, oxidative stress damage (MDA and AOPP) increased following CR exercise and 15/15 intermittent running exercise, and antioxidant defense enzymes (SOD) increased following the CR exercise and decreased following 30/30 intermittent running exercise. These findings indicate the development of oxidative stress with running exercise and are in agreement with previous studies demonstrating an increase of oxidative stress parameters with different running exercise modalities (aerobic, anaerobic, intermittent, and continuous exercises) [29,30]. However, it is still difficult to interpret how exercise modalities could affect the degree of oxidative stress. Indeed, previous studies assessing the effects of different exercise protocols on oxidative stress markers revealed controversial results. In fact, Ashton et al. [31] and Bailey et al. [32] noted a significant increase in oxidative stress parameters (MDA) following moderate (70% of maximal oxygen consumption) and maximal (time to exhaustion) cycling exercises. However, other studies reported no changes in MDA levels [33,34].

In the present study, MDA levels following the continuous running exercise and 30/30 intermittent running were higher compared to the 15/15 intermittent exercise. This result is not in line with thatreported by Välimäki et al. [35], where theyrevealed that MDA levels were higher during 40 min of intermittent running (2 min running/2 min rest) at 80% of VO_2max_ compared to continuous exercise (40 min at 80% of VO_2max_). The difference between the results of the present study and those of Välimäki et al. [35] could be explained by the difference in intensity and duration of exercise, as well as the characteristics of the participants (e.g., age, nutrition, physical fitness level). We speculate that the observed difference in MDA level between the three running exercises in our study could be referred to as the difference in oxygen consumption in each exercise, as the free radicals’ damage increases proportionally with the increase in oxygen consumption.

Concerning AOPP, the results showed that the AOPP level following continuous running exercise and 15/15 intermittent running were higher compared to the intermittent 30/30 running exercise. This finding contradicts the findings of Kabasakaliset al. [36] who found no differences in carbonyl proteins (i.e., protein oxidation marker) between 2000 m of continuous swimming exercise and 6 × 50 m maximal intermittent swimming exercise. This divergence between the results could be explained by the difference in the markers (AOPP vs. carbonylated proteins), as well as the physical fitness of the participants. However, in the present study and regarding MDA levels, it is difficult to explain the higher PC level in 30/30 intermittent exercise compared to 15/15 intermittent exercise and continuous running exercise. We hypothesize that PC oxidation is less affected with long-time intermittent exercise (30/30) compared to short-time intermittent (15/15) or continuous running exercise. Further investigation assessing other oxidative stress damage markers (e.g., F2-isoprostanes, 8-OHdG) are required in order to clarify the possible link between exercise modality and oxidative stress damage.

Regarding antioxidant defenses, the present study results showed a variation in post-exercise SOD activity depending on the exercise modality. Indeed, SOD activity increased after continuous running exercise, remained unchanged after 15/15 intermittent exercise, and decreased after 30/30 intermittent running. This difference in SOD activity could be referred to the difference in FR production between the three exercise modalities. It seems that continuous running has induced a major production of superoxide radical (O_2_^−^). In consequence, SOD activity, the first line of antioxidant defenses, increased to protect from the deleterious effects of O_2_^−^ radicals [37]. Concerning 15/15 intermittent exercise, it seems that the balance between FR and antioxidants remains stable with this type of exercise and that the majority of the O_2_^−^ radicals produced are neutralized continuously by existing antioxidant defenses, which could explain the lack of variation in SOD activity [38]. However, in 30/30 intermittent exercise, the decrease in SOD activity following the exercise could occur besides using enzyme during the dismutation of O_2_^−^ radicals. It seems that for this type of exercise, the O_2_^−^ production was not high enough to induce a SOD reaction [39]. Further studies evaluating FR production are required to clarify the mechanisms for the changes in, and interplay between, exercise modality and antioxidant biomarkers.

GPX activity did not change after running exercise in all three experimental sessions. This finding is in agreement with Jimenez et al. [40] who noted that the concentration of GPX remains unchanged after a maximal cycling exercise in sedentary subjects. The authors explained the lack of changes inGPX values by the fact that the duration of exercise would not have been long enough to generate a high level of hydrogen peroxide (H_2_O_2_) ions and subsequently induce a change in GPX activity. In the present study, and considering the results of SOD after exercise, it seems that the majority of O_2_ radicals produced by exercise were neutralized by SOD, the first line of antioxidant defenses. As a result, only a few H_2_O_2_ radicals were formed, which could explain the lack of alterations in GPX activity following the three running exercise protocols. Finally, it is important to mention that a lack of changes to the antioxidant enzymes does not reflect the absence of FR production, as many other potential sources of FR generation, including endothelial cells (via xanthine oxidase and/or NADPH oxidase), activated leukocytes (via NADPH oxidase), the ischaemia–reperfusion phenomenon, and oxidation of haemoglobin could also contribute to increased production of FR during the exercise, and therefore allow foroxidative stress damage [41].

### Experimental Considerations

The findings of the present study indicate that exercise modality might be a key determinant of oxidative stress perturbation evoked by exercise, where some limitations inherent to the experimental protocol of the present study warrant mention. First of all, although the MDA was considered as the marker mostly used in the literature to characterize lipid peroxidation, other markers, such as F2-isoprostane and hydroperoxydes lipids (LOOH), would have provided stronger data on lipid peroxidation. In addition, the evaluation of FR production would give us a better idea of the evolution of the oxidative stress markers according to the exercise modality. Therefore, drawing several blood samples after exercise could provide more accurate data to characterize the oxidative stress response following exercise. Secondly, oxygen consumption was not analyzed during the experimental sessions. Thus, a control of oxygen consumption, using a portable gas analysis system, for example, would have consolidated the results of the present study.

## 5. Conclusions

The aim of the present study was to determine the effect of running exercise modality on oxidative stress biomarkers. Based on the results of MDA, we can conclude thatcontinuous or 15/15 intermittent exercise induced less radical damage compared to 30/30 intermittent exercise. On the other hand, the results obtained allow us to conclude that the modality of running exercise determines the antioxidant response. In comparison with continuous exercise or intermittent 30/30 exercise, intermittent 15/15 exercise promoted a better balance between free radicals and antioxidants. From a practical view, during endurance training sessions, it is recommended for coaches and athletes to adopt an intermittent 15/15 running exercise session in order to avoid free radical damage resulting from continuous exercise.

## Figures and Tables

**Table 1:** Total daily energy intakes, and macronutrient and antioxidant micronutrient consumption during the period of the three different running exercise modalities (mean ± SD).

	Continuous Running	15/15 Intermittent Running	30/30 Intermittent Running
**Energy intake (Mj/day)**	12.4 ± 1.7	13.2 ± 2.1	11.9 ± 2.3
**Carbohydrates (g/day)**	328.4 ± 77	320 ± 32.1	294.7 ± 49.3
**Proteins (g/kg(BW)/day)**	2.16 ± 0.3	2.1 ± 0.3	1.9 ± 0.9
**Lipids (g/day)**	108.5 ± 37.5	109 ± 33.1	111.2 ± 24.3
**Vitamin C (mg/day)**	103.2 ± 17.8	109 ± 20.1	101.4 ± 27.1
**Vitamin E (mg/day)**	11.8 ± 2.2	12.9 ± 2.1	10.5 ± 3.6
**Selenium (µg/day)**	69.1 ± 19.5	75.1 ± 6.6	74.1 ± 19.2
**Zinc (mg/day)**	13.1 ± 2.7	13.0 ± 2.8	11.6 ± 4.1
**Copper (mg/day)**	1.5± 0.2	1.9 ± 0.3	1.1 ± 0.8

Mj/day: megajoule/day. BW: body weight.
